# Supplementary material for: An Entropy-Based Approach to Measuring Diet Adherence
Source: Entropy (Basel). 2023 Aug 24;25(9):1258. doi: 10.3390/e25091258 (PMC10528816; doi:10.3390/e25091258)
Supplement: Supplementary file 1 [file entropy-25-01258-s001.zip › Table S1.pdf]

Supplemental material

Table S1: Classification of food items based on the Mexico City's normative basket. The table also shows the food items included in the analysis that were available in the Mexican National Survey of Household Income and Expenditure 2018

		ENIGH 2018	Mexico City's normative food basket
**Food groups**	**Subgroups**	**Food items**	**Food items**
GRAINS	Rice	Rice	Rice
	Oats	Breakfast cereal (maize, wheat, rice, oats, granola)	Oats
	Maize		Maize breakfast cereal (no sugar added)
		Corn tortilla	Corn tortilla
	Wheat	White bread	White bread
		n/a	Wheat bread
		Pasta	Pasta
ROOTS AND TUBERS		Potato	Potato
LEGUMES		Beans	Beans
		Lentils	Lentils
NUTS AND SEEDS		Bulk seeds (including peanuts)	Toasted peanuts
VEGETABLES	Fresh vegetables	Green leaves (chard, spinach, purslane)	Spinach
		Zucchini	Zucchini
		Onion	Onion
		Chayote squash	Chayote squash
		Tomato	Tomato
		Cactus	Cactus
		Chili	Chili
		Green tomato	Green tomato
		Carrot	Carrot
FRUITS	Fresh fruits	Lemon	Lemon
		Apple	Apple
		Cantaloupe	Cantaloupe
		Orange	Orange
		Papaya	Papaya
		Banana	Banana
PROCESS MEAT	Processed meat	Pork ham	Pork ham
MEATS	Chicken	Chicken (legs, thighs, breast)	Chicken (thighs and legs)
	Beef	Beef fillet	Beef

FISH		Canned tuna<br>Fish fillet	Canned tuna (in oil)<br>Fish fillet
DAIRY PRODUCTS	Cheese	Fresh cheese<br>Oaxaca or asadero cheese	Fresh cheese<br>Oaxaca cheese
	Yogurt	n/a	Natural yogurt
MILK		Pasteurized cow milk	Whole milk<br>Semi-skim milk<br>Lactose-free milk
		n/a	Breast milk
		n/a	Substitute to breast milk
EGGS		Chicken egg	Egg
FAT AND OILS		Vegetable oil<br>Avocado	Oil<br>Avocado
SUGARS		White and brown sugar	Brown sugar
